# Transgender Associations and Possible Etiology: A Literature Review

**DOI:** 10.7759/cureus.1984

**Published:** 2017-12-24

**Authors:** Fatima Saleem, Syed W Rizvi

**Affiliations:** 1 Internal medicine, King Edward Medical University Lahore, Pakistan; 2 R Endocrinology, New Jersey, Asst. Professor, Internal Medicine and Endocrinology, Umdnj

**Keywords:** transgender, gender identity disorder, gender dysphoria, transexualism, autism spectrum disorders

## Abstract

Transgender or gender dysphoria has been defined in the fifth edition of the Diagnostic and Statistical Manual of Mental Disorders (DSM-5), as distress resulting from the incongruence between one’s experienced gender and one’s assigned gender, along with a persistent and strong desire to be of another gender, and accompanied by clinically significant distress. Adolescents referred for evaluation often want hormonal therapy and several among them also express a desire for gender reassignment surgery. Furthermore, evidence shows that adolescents and adults with gender dysphoria without a sex development disorder, before gender reassignments, are at increased risk for suicide. For this review, a search of the English language scientific literature was conducted using the PubMed database. This summary discusses the associations and comorbidities of gender dysphoria and reiterates the evidence that its etiology is multifactorial. Transsexualism involves prenatal neuroanatomical changes, has a psychiatric association, and is found to be more prevalent in conjunction with schizophrenia and autism spectrum disorders. Childhood adversities and neglect are also linked to having a transgender identity. Moreover, the evidence favors a genetic predisposition. Likewise, there seems to be a growing concern with regards to the relationship between endocrine disruptors and transsexuals as well as other gender minority populations. More research needs to be done to understand the exact pathways.

## Introduction and background

Transgender or gender dysphoria (GD) has been defined as clinically significant distress resulting from the incongruence between one’s experienced gender, and one’s assigned gender, along with a persistent and strong desire to be of another gender [1]. Adolescents referred for evaluation often request hormone therapy (HT) and many also express a desire for sex reassignment surgery (SRS). Evidence shows that adolescents and adults with GD without a sex development disorder before SRS are at increased risk for suicide. However, after SRS, the adjustment may vary, and suicide risk may persist [1]. GD has replaced the term gender identity disorder (GID) in the 2013 edition of the diagnostic and statistical manual of mental disorders, fifth edition (DSM-5), which itself has replaced “transsexualism” [2].

The transgender community suffers from a lot of discrimination. In a survey in the United States, 19% of transgender people reported being denied medical care. Nearly half of those surveyed reported having to teach their medical providers how to care for the transgender persons [3]. It is imperative to perform more research regarding transgender status and associations in order to reveal the complex multifactorial etiology, improve the understanding among clinicians, and lead to better wellbeing for transgender individuals. GD involves neurodevelopmental changes and has psychiatric association including schizophrenia and autism. Childhood adversities are also associated with being transgender. Moreover, the evidence favors a genetic predisposition. A link with endocrine disrupting chemicals has also been suggested.

There are several transgender variants that exist historically in different cultures of the world [4]. They do not fit in the conventional definition of male or female; rather, they move between the two or are a combination of both genders. Vulnerable and insecure transgender individuals have been disregarded and marginalized by the mainstream society [4]. In a study in Bangladesh, 50 in-depth interviews and 20 key-informant interviews were conducted. Results revealed that transgender persons suffered from humiliation at school, social isolation, and difficulty getting a mainstream job. Moreover, family members of transgender persons felt uncomfortable with the feminine attitudes of male adolescents, especially when the family experienced negative and unpleasant societal expressions [4].

In the United States, individuals who identify as transgender experience similar adversities. A study including transgender and gender non-conforming individuals from all fifty states showed the presence of discrimination against transgender people [3]. Transgender people were living in extreme poverty. Forty-one percent of them reported attempting suicide as compared to 1.6% of the general population. Those who expressed a transgender identity or gender non-conformity at schools reported harassment (78%), physical assault (35%), and sexual violence (12%). About one-sixth left school or higher education due to harassment. Of those who have transitioned to the desired gender, only 59% reported updating the gender on their driver’s license or state identity documentation [3]. Likewise, human immunodeficiency virus (HIV) infection was found to be more prevalent in transgender community as compared to the general population. Fifty-seven percent of transgender persons experienced significant family rejection. Seventy-six percent have been able to receive HT, demonstrating their determination, ability to endure difficulties, and resolution to seek out sensitive medical providers. Over three-fourths reported feeling more comfortable at work and improved performance after transitioning, although these transgender people had the same rates of harassment at work as the general population [3].

In another survey conducted in medical students enrolled in the United States and Canada, 15.8% students from 152 institutions identified themselves as sexual and gender minorities. The most common reasons for concealing their sexual identity were privacy, fear of discrimination, and social norms [5]. Research in the United States on transgender people has mostly relied on convenience samples taken from urban HIV assessments as most of the health surveillance surveys do not have measures that allow transgender identification [6]. Conron, et al. indicated that transgender adults were more likely to be unemployed as well as living at less than or equal to 100% of the poverty index as compared to non-transgender individuals among the survey participants selected from the Massachusetts Behavioral Risk Factor Surveillance System. Also, transgender people are less likely to be overweight, but more likely to smoke [6]. When transgender people were compared to their non-transgender siblings, it was concluded that even when transgender and gender queer people are more highly educated in comparison to their non-transgender siblings, they do not have a corresponding increase in their income. Transgender people are more likely to experience harassment and discrimination than their non-transgender siblings. They have less social support from family than non-transgender individuals [7].

In 2014, the behavioral risk factor surveillance system (BRFSS) included an optional gender identity module and was adopted in nineteen states in the United States [8]. Transgender people’s demographics from BRFSS data show that they make up to 0.53% of the population. They are more likely to be non-white and below the poverty line, as likely to be married (50.5% vs. 47.7%), living in a rural area (28.7% vs. 22.6%), less employed, and less likely to attend college (35.6% vs. 56.6%) as compared with the non-transgender individuals [8]. A larger proportion of individuals identify themselves as male-to-female rather than female-to-male or gender nonconforming. Overall, transgender respondents are not significantly different from the non-transgender population with regards to age, living in a rural area, marital status, or employment [8]. Another study to identify the transgender population in the United States based on Behavioral Risk Factor Surveillance System (BRFSS) data showed 0.6% adults (about 1.4 million) identifying themselves as transgender people [9]. Hawaii has the highest percentage occurring in 0.8% of adults, while North Dakota has the lowest percentage at 0.3%. The district of Columbia (DC) was not included in this dataset. DC has a notably high transgender percentage (2.8%) and is considered an outlier [9].

## Review

Given the demographics and background of transgender people, a growing transgender population invites researchers to dig further into the etiology and comorbidities related to transgender people in order to improve the overall health of the transgender community.

Data sources

The review covers publication dates within the last ten years. A search of English scientific literature was conducted using PubMed. Studies involving transgender demographics, discrimination, transgender etiology, and associations were included. 

Neuroanatomical etiology

Differences in the brain structures and brain functions that are related to gender and sexual orientation have been found to be associated with the pathoetiology of gender dysmorphic disorder. Sexual differentiation of the genitals takes places in the first trimester of pregnancy, and sexual differentiation of the brain starts during the second half of pregnancy. Therefore, it has been hypothesized that these two processes may play roles independently of each other that predispose an individual to transsexuality [10]. One working hypothesis behind GD is that the neuronal differentiation in the hypothalamic networks is altered [11]. Magnetic resonance imaging (MRI) of male-to-female transsexual conversion has shown a female-like putamen; this means that the transgender putamen has a volume that is larger than normal males but within the normal female range [12]. Combined positron emission tomography and MRI experiments have shown sexual dimorphism in hemispheric ratios and the pattern of amygdala connectivity [13]. Sex reversal of the INAH3, a subnucleus of the hypothalamic uncinate nucleus in transsexual people, is a probable marker of an early atypical sexual differentiation of the brain. Changes in INAH3 and the bed nucleus of the stria terminalis (BNST) may belong to a complex network that may structurally and functionally be related to normal sex differentiation [14].

Psychiatric associations

GD patients appear to have comorbid psychiatric disorders, most commonly anxiety and depressive disorders [1]. Studies done in Amsterdam, Ghent, Hamburg, and Oslo have shown that 70% of the individuals with GD are diagonised with lifetime DSM-IV-TR Axis I; mostly, affective disorders and anxiety problems [15]. In a survey in Iran, major depressive disorder (33.7%), specific phobia (20.5%), and adjustment disorder (15.7%) were found to be the three most prevalent disorders in GD patients requesting SRS. Bipolar mood disorder prevalence was 2.4%. The majority of the patients with GD were found to have psychiatric Axis I comorbidities [16]. The literature shows that, although psychiatric conditions are more prevalent, there are low rates of coexistent medical illness in GD. Depression is present in 34.4% of the patients. Moreover, transgender people are much less likely to attend a mental health professional [17].

HT in transgender people has been found to boost self-esteem, reduce depression, and improve quality of life [18]. Initiating HT in transgender people seems to have a positive effect in reducing stress levels as shown by the reduced cortisol levels, assessed by cortisol awakening response, measured before and twelve months after HT [19]. Likewise, there is a decrease in depression but slightly elevated anxiety in transgender children who have socially transitioned and are supported to live openly. These children now identify themselves as the gender “opposite” to their natal sex [20]. Studies also show a correlation of GD with eating disorders [21]. Further studies need to be done to further substantiate these claims. 

Relation with autism spectrum disorder

Autism spectrum disorder (ASD) is more prevalent in GD than in the general population [1]. Evidence suggests a link between GD and ASD [22]. To study further, additional studies with larger sample size are needed [2]. Evidence shows that 5.5% of the GD patients showed ASD traits as compared to the general population [23]. The literature evidence as regards to the co-occurrence of ASD and GD is limited. This is important as paying attention to the development of gender identity formation in individuals with ASD from an early age may be helpful. Doctors should also help individual to explore his or her own gender narrative, rather than merely focusing on medical intervention [24].

Schizophrenia

There is growing evidence, although limited, suggesting that both GD and schizophrenia are neurodevelopmental disorders [25] and that they may share common causal mechanisms and risk factors. Both involve brain lateralization and pathways involved in sexual differentiation [26]. A study performed by Judge, et al. documents presence of schizophrenia in 3.67% patients with suspected or confirmed GD who were referred for HT consideration [17]. A college-based survey in China shows that GD is more prevalent in women. Also, the scale of correlations between compulsion and GD, and between GD and schizophrenia, is significantly greater in male participants [27]. However, not all studies have shown a correlation [16].

Baltieri and De Andrade reported a case of a transgender patient with schizophrenia and the risks of a potential diagnostic confusion. A 19-year-old woman was referred for SRS, having an eight-year history of undifferentiated schizophrenia and GD. After a more suitable antipsychotic treatment, her masculine behavior persisted but her desire to change her own genital organs decreased. The case points out the challenge of differentiation between pure identity disorders and transsexual feelings secondary to an ongoing psychopathologic process such as schizophrenia [28].

Toxoplasma

Rajkumar also observed an association between Toxoplasma infection, schizophrenia and GD based on studies suggesting an independent association of both schizophrenia and GD in prenatal toxoplasma [29-30]. He suggests testing the hypothesis serologically in GD. However, no hard data is available [26].

Childhood maltreatment

A growing body of evidence reports the association between childhood maltreatment and adult dissociative psychopathology [31-33]. In a study [34] performed in Florence, 109 patients meeting the criteria for male-to-female GD were interviewed for childhood maltreatment with regards to emotional abuse, neglect, physical abuse, and sexual abuse. A high proportion of transsexual subjects reported childhood maltreatment. Maltreated subjects also reported higher body dissatisfaction and showed a worse lifetime mental health. Nevertheless, approaching the issue of childhood neglect and maltreatment thoroughly can enable the patients to reflect on its impact on their lives and, eventually, its relevance on treatment decisions [34].

Role of endocrine disruptors

Another working hypothesis involves the role of endocrine disruptors in transgender etiology. In a letter to the editor, Bejerot, et al. suggested a hypothetical link between endocrine disrupting chemicals and transgenders [35]. They hypothesized a role for endocrine disruptors, especially phthalates. Phthalates are present in some plastics, and there has been an increased concentration in the environment in recent years. Bejerot, et al. suggested that endocrine disruptors may be the cause of high fetal testosterone exposure leading to increased risk of ASD as well as GD [35]. More systematic investigations are required that may also elaborate mechanisms involved in brain development and sexual differentiation.

Research in rats has shown that endocrine disruptors like polychlorinated biphenyls (PCB) profoundly impair the sexual differentiation of female hypothalamus [36]. Also, there is a growing concern that the use of fragrance-containing daily lifestyle materials run parallel with the unprecedented rates of malignancies, neural ailments, teratogenicity, and transgender instances [37]. PCB exposure can lead to altered neuronal activity in hippocampus and identify it as potential environmental risk factors for neurodevelopmental disorders. The mechanism involves probable defects in neuronal Ca++ signaling, increased ryanodine receptor activity and dendritic growth in hippocampal neurons caused by PCP [38].

Genetics

Likewise, a genetic association has been proposed. Heritability of GD is suggested by evidence that has shown the familiality of transsexualism among non-twin siblings, and an increased concordance for transsexualism in monozygotic as compared with dizygotic same-sex twins [1]. In addition, research supports CYP17 as a candidate gene for female-to-male transsexualism and shows that loss of a female-specific CYP17 T-34C allele distribution pattern is linked with female-to-male transsexualism [39].

## Conclusions

The literature on transgender people during the last decade provides a framework for the associations and comorbidities associated with transgender or GD. The research draws links between transgender people and changes in prenatal neuroanatomy. There is an association with psychiatric disorders, schizophrenia, and ASD. Transsexualism is linked to childhood maltreatment and adversities. Evidence also leads some to speculate that there are genetic predispositions. Furthermore, a working hypothesis exists with regards to possible association of endocrine disrupting chemicals and transgender identity or other gender-related issues. The evidence until today shows that transsexualism has a complex biopsychosocial etiology. There is a need for additional research to explore the myths and mysteries behind transgender identity to improve the understanding among clinicians, social activists and policy makers leading to better transgender health.
